# Electronic Health Record-Related Burnout: Implementing a Data-Driven Quality Improvement Intervention for Ambulatory Providers After an Electronic Health Record Transition

**DOI:** 10.7759/cureus.113338

**Published:** 2026-07-25

**Authors:** Shadi Hijjawi, Richard Schreiber, Robin Lang, Salim Saiyed

**Affiliations:** 1 Medicine, Penn State University College of Medicine, Milton S. Hershey Medical Center, Hershey, USA; 2 Nursing, CaroMont Health, Gastonia, USA; 3 Internal Medicine, University of Texas at Austin Dell Medical School, Austin, USA

**Keywords:** after-hours, burnout, efficiency, electronic health records, primary care physicians

## Abstract

Background

Electronic health records (EHRs) have improved healthcare delivery but have also introduced workflow inefficiencies that contribute to clinician burnout. After-hours EHR use has been identified as a key contributor, particularly among ambulatory providers.

Objectives

To evaluate the impact of a targeted EHR optimization intervention on self-reported EHR-related burnout and perceived efficiency among ambulatory providers following EHR implementation.

Methods

We conducted a quality improvement interventional study among ambulatory providers after transition to a new EHR. Self-reported EHR-related burnout and user experience outcomes were assessed using pre- and post-optimization surveys. Objective EHR event log data were used to evaluate after-hours EHR activity and efficiency metrics. Descriptive comparisons were performed to evaluate changes in perceived efficiency and after-hours EHR use.

Results

A total of 60/67 (90%) providers participated, with 57/60 (95%) completing the optimization training, and approximately 37% (22/60) were primary care providers (PCPs). Baseline self-reported EHR-related burnout was common. Following the intervention, most providers who initially reported burnout noted improvement, and nearly all participants reported increased efficiency in EHR use. Providers with higher after-hours EHR use were more likely to report burnout, with PCPs reporting greater after-hours EHR burden compared to non-PCPs.

Conclusion

EHR-related burnout remains prevalent among ambulatory providers, particularly those with substantial after-hours EHR use. Targeted EHR optimization interventions may improve perceived efficiency and reduce burnout. Further studies with more robust methodologies are needed to better quantify these effects.

## Introduction

Electronic health records (EHRs) have improved access to clinical information and enabled decision support, but their impact on physician workload and burnout remains debated [1]. While EHR use may reduce certain clerical tasks, it can also increase documentation and administrative burdens for clinicians [2]. Physician well-being and burnout have become major concerns, with reported rates consistently high [3]. A Mayo Clinic study found that 54% of physicians experienced burnout in 2014, with even higher rates among family physicians (63%) [4]. Another study reported that 45.8% of physicians had at least one symptom of burnout [5]. A recent study showed that burnout remains highly prevalent among United States (US) physicians, with primary care physicians experiencing the highest rates across specialties [6]. Burnout is an expensive problem, costing more than $4.6 billion annually in the US [7]. It is characterized by overwhelming exhaustion, feelings of cynicism and detachment, and a sense of ineffectiveness and lack of accomplishment [8].

Recently, there has been more demand to address and improve healthcare providers' work-life, especially physicians, to achieve the quadruple aim, which includes improving patient experience, enhancing population health, reducing healthcare costs, and improving the well-being and work-life of care teams [9]. Some studies highlighted the effect of EHRs on physicians' and other healthcare providers' stress and burnout, including the increased adoption of EHRs in recent years, and the effects of an increasing number of functions and time spent in the EHR, especially after-hours (time spent in the EHR after regular clinic hours, usually 5 pm to 8 am) [6,10-12]. For each hour providers spend in patient care, they spend two hours doing administrative work, mainly in the EHR [13]. Despite that, data about EHR-related burnout, especially the effects of after-hours activity in the EHR, remain limited, including the magnitude of the problem, the efforts to address it, and the strategies to alleviate it. For example, in a meta-analysis, interventional studies addressed physician burnout by discussing various related issues, such as individual and organizational interventions, including mindfulness, stress management, and workload reduction, but they did not address any direct EHR-related interventions [14]. With the growth of EHR implementation and the reported relationship of EHR use to burnout, continuous and personalized training after EHR implementation is effective in reducing the time spent using the system [15]. EHR event log (time-stamped records of user interactions with the EHR) data analytics can identify opportunities for improvement [16-20].

In our study, we provide a data-driven analysis of EHR-related burnout by adopting a strategy of combining subjective and objective data in evaluating ambulatory provider burnout, especially among primary care providers (PCPs). In addition, we offer a novel way to quantify the relationship between EHR after-hours usage and provider burnout, reinforcing the need to optimize EHR use to support provider well-being and efficiency. 

Objectives

The primary objective was to evaluate whether a targeted EHR optimization intervention was associated with changes in self-reported EHR-related burnout and perceived efficiency (survey measures) among ambulatory providers following an EHR transition. Secondary objectives were to estimate the prevalence of self-reported EHR-related burnout and to examine the relationship between after-hours EHR activity, measured using event log data, and burnout.

## Materials and methods

A quality improvement interventional study was performed by the Provider Informatics team at CaroMont Health, a community-based not-for-profit health system in the western Charlotte Metropolitan area in Gastonia, North Carolina. CaroMont Regional Medical Center is a 435-bed tertiary care hospital that serves an immediate community of 220,000 people. The interventional training targeted CaroMont Medical Group (CMG) ambulatory providers (approximately 115 physicians and advanced practice providers (APPs)), including PCPs and specialist providers practicing at 46 clinics. CaroMont Health ambulatory clinics transitioned to a new Epic EHR from Athenahealth in 2016. The intervention program was organized a few weeks after the transition.

This study was conducted as a quality improvement initiative using anonymous, de-identified survey and EHR event log data and does not meet the definition of human subjects research per guidance from the Office for Human Research Protections.

The survey (subjective data)

We evaluated EHR-related burnout by surveying ambulatory providers about perceived burnout and efficiency using the newly adopted EHR (Epic; Epic Systems Corporation, Verona, WI). Pre- and post-optimization surveys were internally designed and not formally validated; however, selected items were informed by prior literature on EHR-related workload and burnout. The surveys were electronically distributed to all participating providers before and after training sessions. An even-point (4-point Likert scale) was used to select answers to survey questions (Strongly Agree, Agree, Disagree, and Strongly Disagree).

The event log (objective data)

Epic's event log-based tool, the Provider Efficiency Profile (PEP; later referred to as "Signal"), was used to evaluate provider efficiency, proficiency, and secondary outcomes of the intervention. The PEP/Signal and associated efficiency/proficiency metrics are proprietary analytics tools developed by Epic Systems Corporation. Efficiency metrics were defined based on vendor-provided benchmarks (Epic Systems Corporation, unpublished internal documentation). The EHR efficiency score reflects time spent in the chart relative to peers, while the proficiency score reflects the use of personalized EHR tools. Both scores range from 1 to 10, with 1 being the lowest and 10 being the highest. To have a targeted approach, we assumed that providers with lower efficiency scores are more likely to benefit from the intervention to help alleviate their burnout. All providers with an efficiency score <5.0 were enrolled, and their proficiency score was also collected.

The intervention

The intervention was implemented over six weeks. Baseline survey responses and PEP metrics were collected immediately prior to initiation of training. Providers then completed individualized EHR optimization sessions over approximately eight to nine hours distributed across the intervention period. Post-intervention surveys and efficiency (PEP) assessments were obtained four to eight weeks after completion of the training sessions. The individual training was done by a provider informaticist working in one-on-one sessions with each provider, utilizing direct in-clinic observations, PEP report data, and personal provider feedback. These sessions focused on optimizing key EHR workflows, including chart review, note documentation through templates and smart tools, order entry and preference lists, inbox and results management, and navigation efficiency techniques, such as shortcuts and streamlined chart review workflows.

Our in-class structured training plan was inspired by Stanford Children's Health's design for an individualized EHR learning plan for providers' efficiency [21].

Statistical analysis

Descriptive statistics were used to summarize survey responses and EHR usage metrics. Paired and unpaired comparisons were performed using Student's t-test where appropriate. Associations between categorical variables were explored using contingency tables and chi-square testing. Given the small sample size and study design, analyses are exploratory and intended to identify trends rather than establish causality. Statistical analyses were performed using Statistical Product and Service Solutions (SPSS, version 27.0; IBM SPSS Statistics for Windows, IBM Corp., Armonk, NY).

## Results

There were 67 providers (58%) (out of 115 ambulatory CMG providers) with low efficiency scores (<5.0). Of these, 60 (90%) were successfully enrolled in the intervention, and 57 (95.0%) completed the training intervention (see Table 1 for details). The average enrollment efficiency score for providers was 3.5 (median: 4.1, range: 0-5). Internal Medicine and Family Medicine constituted one-third of the providers' specialties. Specialties of other providers included pediatrics, cardiology, neurology, obstetrics and gynecology, general surgery, infectious disease, cardiothoracic surgery, and vascular surgery. The Provider Informatics training team completed 347 one-on-one sessions with the providers. This included approximately 500 hours with an average of five to six sessions per provider at an average of 8-9 hours per provider.

**Table 1:** Characteristics of the enrolled providers (n=60) PCPs, primary care providers; IM, internal medicine; FM, family medicine

Characteristic	No. (%)
Mean age in years	47 (SD 8.9)
Female	25 (41.6)
Completed intervention	57 (95.0)
Physician	40 (66.6)
PCPs (IM and FM)	22 (36.6)
Advanced care practitioner	20 (33.3)
Mean years of practice	14.1 (SD 8.9)

Of the 57 providers, 41 (72%) responded to the pre-optimization survey, and 37 (65%) responded to the post-optimization survey (Table 2). Self-reported EHR-related burnout affected 54% (22/41) of the ambulatory providers. Of those, 50% (11/22) represented internal medicine and family medicine PCPs. Nearly 51% (21/41) spent 60 minutes or more in the EHR outside regular clinic hours (8 am to 5 pm). All providers (41) except one (98%, n=40/41) felt the optimization sessions were a good use of their time and recommended that they be extended to their colleagues.

**Table 2:** Subjective outcomes of optimization EHR, electronic health record; PCP, primary care provider

Outcome	n/N (%)
Pre-optimization survey (n=57)	
Response rate	41/57 (71.9)
Spent >60 minutes in the EHR after-hours	21/41 (51.2)
Self-reported EHR-related burnout	22/41 (53.7)
PCP reported EHR-related burnout	11/22 (50.0)
Post-optimization survey (n=57)	
Response rate	37/57 (64.9)
Providers not reporting burnout	11/37 (29.7)
Providers reporting burnout	26/37 (70.3)
Providers reported improvement in burnout	19/26 (73.1)
Providers reported more confidence using EHR	36/37 (97.3)

After finishing optimization sessions, 73% (19/26) of respondents with baseline burnout reported perceived improvement. The majority (73-89%, n=27-33/37) also reported improvement in their efficiency across different EHR functions, including clinical chart and inbox review, note templates and documentation, and ordering functions (Table 2).

Efficiency and proficiency scores and after-hours activity in the EHR were compared before and after intervention for all providers who underwent training (n=57) and who continued to practice at the time of reevaluation (91%, n=52/57). Efficiency scores increased for 56% (29/52) of the providers, while proficiency scores increased for 69% (36/52) after the intervention. There was better improvement in proficiency compared to efficiency scores. The average efficiency score before optimization slightly improved from 3.7 to 3.9 after optimization (p=0.13). The average proficiency score improved from 6 to 7 (p<0.01). As part of the data analytics provided, the time spent in the main clinical EHR functions was calculated for all providers. Overall, after comparing the time before and after the intervention, there was an average of at least 1.26 minutes of reduced time in the EHR after the intervention for each patient or encounter for each provider (Table 3).

**Table 3:** Average time (minutes) spent by each provider per patient or encounter in the main clinical EHR functions* EHR, electronic health record. *p-Value=0.05. ^†^All values are positive, as the time spent was less after optimization in all functions.

EHR function	Before optimization	After optimization	Time reduced after optimization^†^
Chart review	3.01	2.75	0.26
Ordering	3.67	3.56	0.11
Documentation	6.85	5.96	0.89
Overall time	13.53	12.27	1.26

After-hours or “late” activity in the EHR highlights time spent in the EHR while at home (also called Pajama Time) after regularly scheduled office hours (8 am-5 pm). Before optimization, the group of enrolled providers was staying late on average 8.4 days per provider per 15 business days of event log monitoring. After optimization, this decreased to an average of 5.2 days per provider, a 38% ((5.2-8.4)/8.4 days) reduction (p<0.01). The average time spent (in minutes) after-hours per scheduled day was 156.3 minutes before the intervention, decreasing to 129.3 minutes after. Despite this apparently dramatic reduction (27 minutes) in time spent after-hours, it was not statistically significant (p=0.52).

We used 2x2 contingency tables to evaluate the association between after-hours activity and self-reported burnout stratified by provider specialty. We considered a clinically significant after-hours activity to be any time equal to or longer than 60 minutes, whether obtained from data logs or as subjectively estimated on the pre-survey. More than two-thirds (68%, n=15/22) of all providers reported having burnout using the EHR reported spending more than 60 minutes in after-hours activity (5 pm-8 am) (Table 4).

**Table 4:** Association between subjective information of spending clinically significant time in after-hours activity in the EHR and self-reported burnout EHR, electronic health record. *Two-thirds are primary care providers. ^†^n with available data from pre-optimization survey.

After-hours activity	No. (%)		
	Reported burnout	No burnout	Total
>60 minutes	15 (68.2)*	6 (31.6)	21 (51.2)
<60 minutes	7 (31.8)	13 (68.4)	20 (48.8)
Total	22 (100)	19 (100)	41 (100)^†^

On the other hand, the association between after-hours activity from data logs and the specialty of the provider showed that 84% (16/19) of internal medicine and family medicine providers spent a significant amount of time after-hours. Most of the other specialist providers (88%, n=29/33) spent much less time after-hours (Table 5). There was no observed association between self-reported EHR-related burnout and being a PCP as evaluated from the pre-optimization survey data.

**Table 5:** Association between objective data of spending clinically significant time in after-hours activity in the EHR with being an IM or FM provider EHR, electronic health record; IM, internal medicine; FM, family medicine. ^†^n with available data post-optimization.

Duration of after-hours activity	No. (%)		
	IM or FM provider	Other providers	Total
Significant after-hours (>60 minutes)	16 (84.2)	4 (12.1)	20 (38.5)
Less significant after-hours (<60 minutes)	3 (15.8)	29 (87.9)	32 (61.5)
Total	19 (100)	33 (100)	52 (100)^†^

## Discussion

This study does not establish a causal relationship between EHR use and burnout but rather explores associations between EHR-related workload metrics and self-reported burnout in the context of a targeted optimization intervention.

Self-reported burnout among a selected group of ambulatory providers is not different from the national averages, but we correlate it directly to EHR use. A comprehensive approach to reduce clinician burnout as per the National Academy of Medicine includes the following: “to enable technology solutions, provide support to clinicians and students and invest in research” [22]. Therefore, we started our intervention by seeking feedback from our providers as a key factor to succeed in this quality improvement project after a new EHR transition.

EHR audit trail logs are a valid source for clinical workflow analysis, as they provide a simple method for healthcare systems to examine how their clinicians spend their time [17,18,23]. Our study is one of a few similar studies that highlight an effective utilization of such data log systems. Although sometimes they are not completely accurate and may need further improvement by EHR vendors, the data driven from these systems can be extremely helpful if analyzed in the appropriate context [23]. We found that provider time spent in the EHR is hard to improve since it can vary daily and is dependent on individual learned behavior during clinical practice. In contrast, personalization sessions can improve proficiency scores [24,25]. We believe that reviewing objective data from data logs with each provider is the key to improving their efficiency by having insight into their “blind spots”. In a multi-institutional survey, the Arch Collaborative (KLAS) study found that physicians who update or personalize their settings are more than twice as likely to be highly satisfied with their EHR [26]. 

Data found that providers devote 1-2 hours on EHRs and administrative work for every hour spent in direct patient care. Additionally, they spend 1-2 hours of personal time after office hours [13,16,17]. A study showed the average time spent by ambulatory providers is 16 minutes per encounter using the EHR. This time varies between specialties and the different clinically focused EHR functions [27]. In Table 3, we showed similar comparable information with less average overall time in the three major clinical EHR functions, especially after optimization. After optimization, each provider reduced an average of 1.26 minutes per patient, and since the average family physician sees 19 patients per day [28], this suggests that each provider could save up to 24 minutes per day in the EHR. If we extrapolate this time over a year (average of 200 workdays), then each provider can save an average of 80 hours in the EHR per year. That is a total of 10 business days less time in the EHR. Similar work demonstrated the same concept of the effect of optimization programs [29]. This time savings may help prevent burnout and empower the provider to deliver high-quality, safe patient care.

These findings suggest that PCPs often spend more time in the EHR after-hours, but our small sample was limited and may not fully capture all contributors to burnout. Although we observed associations between after-hours EHR activity and perceived burnout, no clear association was observed between being a PCP and self-reported burnout in our subjective data.

The study has several limitations. It lacks a control group, limiting attribution of observed changes to the intervention. The targeted inclusion of providers with low baseline efficiency introduces potential regression to the mean. The sample size is small with incomplete survey participation, limiting generalizability. Burnout was assessed using a non-validated, single-item survey measure focused on perceived EHR-related burnout, which limits comparison with studies using validated burnout instruments. The pre-post design does not account for temporal trends such as learning curve effects following EHR implementation or concurrent system updates. Time evaluation in log data focused on major sections and may underestimate time spent in other EHR tasks and did not identify the specific activities contributing to after-hours work (e.g., documentation, inbox management, or chart review). Data were collected a few years ago at a single health system, which may limit applicability to current practice and other settings.

## Conclusions

In conclusion, this study suggests that PCPs tend to spend significant time in the EHR after-hours and may be more likely to report EHR-related burnout following a transition to a new EHR. Tailored optimization interventions that combine subjective feedback and objective usage data may help improve efficiency and reduce perceived EHR-related burden. However, larger, controlled studies are needed to confirm these findings and to guide strategies for supporting clinician well-being. All healthcare institutions should start their initiatives to address EHR-related burnout by using well-designed strategic intervention plans. A good strategy to maintain physicians' and other healthcare providers' well-being is to continue to measure their EHR satisfaction as the burnout problem continues to grow.

## Appendices

Pre-optimization survey questions

1. I feel confident in my proficiency to use Epic effectively and to quickly perform most tasks

-> Strongly Agree, Agree, Disagree, Strongly Disagree

2. I would benefit from more training to optimize my use of Epic

-> Strongly Agree, Agree, Disagree, Strongly Disagree

3. Epic has contributed to my burnout during my current day-to-day work

-> Strongly Agree, Agree, Disagree, Strongly Disagree

4. I feel standardizing templates and workflows across my specialty or clinic will help with efficiency in Epic

-> Strongly Agree, Agree, Disagree, Strongly Disagree

5. I am using most of Epic functions (Chart Review, Notes, Orders, Inbasket, etc.) appropriately to navigate through my workflow

-> Strongly Agree, Agree, Disagree, Strongly Disagree

6. I need more training to optimize and become efficient in tasks related to the following: (Finding information and reviewing chart)

-> Strongly Agree, Agree, Disagree, Strongly Disagree

7. I need more training to optimize and become efficient in tasks related to the following: (Office visit, consultation & procedure note templates)

-> Strongly Agree, Agree, Disagree, Strongly Disagree

8. I need more training to optimize and become efficient in tasks related to the following: (Documenting problem lists)

-> Strongly Agree, Agree, Disagree, Strongly Disagree

9. I need more training to optimize and become efficient in tasks related to the following: (Ordering medications, labs, imaging, referrals)

-> Strongly Agree, Agree, Disagree, Strongly Disagree

10. I need more training to optimize and become efficient in tasks related to the following: (Health maintenance, dashboard, and quality measures documentation)

-> Strongly Agree, Agree, Disagree, Strongly Disagree

11. I need more training to optimize and become efficient in tasks related to the following: (InBasket workflows such as Results, MyChart, Refill requests)

-> Strongly Agree, Agree, Disagree, Strongly Disagree

12. Approximately, how much time do you spend working in Epic outside clinic hours?

-> 1-10 minutes, 11-30 minutes, 31-60 minutes, 61-120 minutes, >120 minutes

13. Please FREE TEXT other issues and opportunities that you think will help your optimization plan

-> Free text answer

Post-optimization survey questions

1. After optimization sessions, I feel more confident in my proficiency to use Epic effectively and to quickly perform most tasks.

-> Strongly Agree, Agree, Disagree, Strongly Disagree

2. I think I still would benefit from more training sessions to optimize my use of Epic.

-> Strongly Agree, Agree, Disagree, Strongly Disagree

3. Optimization training helped alleviate my EHR-related burnout during my day-to-day work.

-> Strongly Agree, Agree, Disagree, Strongly Disagree

4. After optimization sessions, I am using most of Epic functions that I need (Chart review, Notes, Orders, In-Basket, etc) more appropriately to navigate through my workflow.

-> Strongly Agree, Agree, Disagree, Strongly Disagree

5. I have significant improvement in my efficiency in tasks related to the following: (Finding information and reviewing a chart)

-> Strongly Agree, Agree, Disagree, Strongly Disagree

6. I have significant improvement in my efficiency in tasks related to the following: (Office visit, consultation, & a procedure note templates)

-> Strongly Agree, Agree, Disagree, Strongly Disagree

7. I have significant improvement in my efficiency in tasks related to the following: (Documenting problem lists)

-> Strongly Agree, Agree, Disagree, Strongly Disagree

8. I have significant improvement in my efficiency in tasks related to the following: (Ordering medications, labs, imaging, referrals)

-> Strongly Agree, Agree, Disagree, Strongly Disagree

9. I have significant improvement in my efficiency in tasks related to the following: (Health maintenance, dashboard and Quality measures Â documentation)

-> Strongly Agree, Agree, Disagree, Strongly Disagree

10. I have significant improvement in my efficiency in tasks related to the following: (In-basket workflows such as Results, Mychart, Refill requests)

-> Strongly Agree, Agree, Disagree, Strongly Disagree

11. I recommend optimization sessions to other providers, as they were a good use of my time.

-> Strongly Agree, Agree, Disagree, Strongly Disagree

12. I need an additional 1:1 training session.

-> Strongly Agree, Agree, Disagree, Strongly Disagree

13. If you agreed to the previous question, what topic(s) would you like to be covered?

-> Free text answer

14. If you have any additional comments about the PEP program\optimization sessions, please provide them below.

-> Free text answer
